# Racemic 2′-hydroxy-4′,4′-dimethylpyran-1,5-dihydroxyxanthone monohydrate

**DOI:** 10.1107/S1600536813021223

**Published:** 2013-08-21

**Authors:** Nawong Boonnak, Suchada Chantrapromma, Hoong-Kun Fun

**Affiliations:** a Faculty of Traditional Thai Medicine, Prince of Songkla University, Hat-Yai, Songkhla 90112, Thailand; b Department of Chemistry, Faculty of Science, Prince of Songkla University, Hat-Yai, Songkhla 90112, Thailand; c X-ray Crystallography Unit, School of Physics, Universiti Sains Malaysia, 11800 USM, Penang, Malaysia; d Department of Pharmaceutical Chemistry, College of Pharmacy, King Saud University, PO Box 2457, Riyadh 11451, Saudi Arabia

## Abstract

The title xanthone (systematic name: 3,6,11-trihy­droxy-1,1-dimethyl-2,3-di­hydro­chromeno[2,3-*f*]chromen-7-one monohydrate), known as pruniflorone N, crystallized as a monohydrate, C_18_H_16_O_6_·H_2_O. The three ring systems of the xanthone skeleton are approximately coplanar, with an r.m.s. deviation of 0.0270 (1) Å from the plane through the 14 non-H atoms. The O atoms of the two hy­droxy substituents on the benzene rings also lie close to this plane, with deviations of 0.019 (1) and 0.070 (1) Å. The 2′-hy­droxy-4′,4′-di­methyl­pyran ring is disordered over two positions with a 0.798 (3):0.202 (3) site-occupancy ratio. An intra­molecular O—H⋯O hydrogen bond generates an *S*(6) ring motif. In the crystal, the xanthone and water mol­ecules are linked into a three-dimensional network by O—H⋯O hydrogen bonds and weak C—H⋯O inter­actions. π–π inter­actions, with centroid–centroid distances of 3.5982 (7), 3.6081 (7) and 3.6456 (7) Å, are also observed.

## Related literature

For details of hydrogen-bond motifs, see: Bernstein *et al.* (1995 ▶). For ring conformations, see: Cremer & Pople (1975 ▶). For bond-length data, see: Allen *et al.* (1987 ▶). For background to xanthones and their biological activity, see: Boonnak, Karalai *et al.* (2010 ▶); Boonnak, Khamthip *et al.* (2010 ▶); Gopal­a­krishnan *et al.* (1997 ▶); Ho *et al.* (2002 ▶); Obolskiy *et al.* (2009 ▶). For related structures, see: Boonnak *et al.* (2006 ▶); Boonnak, Chantrapromma *et al.* (2010 ▶). For the stability of the temperature controller used in the data collection, see: Cosier & Glazer, (1986 ▶).
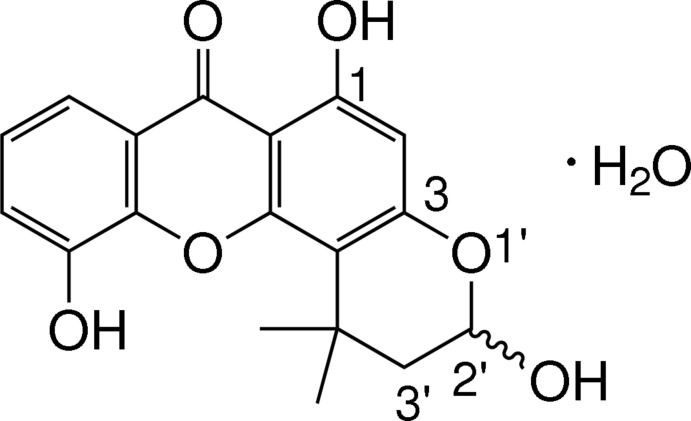


## Experimental

### 

#### Crystal data

C_18_H_16_O_6_·H_2_O
*M* *_r_* = 346.20
Orthorhombic, 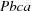

*a* = 9.8965 (2) Å
*b* = 15.2329 (3) Å
*c* = 20.1122 (4) Å
*V* = 3031.96 (10) Å^3^
*Z* = 8
Mo *K*α radiation
μ = 0.12 mm^−1^
*T* = 100 K
0.65 × 0.21 × 0.13 mm

#### Data collection

Bruker APEXII CCD area-detector diffractometer
Absorption correction: multi-scan (*SADABS*; Bruker, 2009 ▶) *T*_min_ = 0.927, *T*_max_ = 0.985
40070 measured reflections
4949 independent reflections
4378 reflections with *I* > 2σ(*I*)
*R*_int_ = 0.030

#### Refinement

*R*[*F* ^2^ > 2σ(*F* ^2^)] = 0.052
*wR*(*F* ^2^) = 0.145
*S* = 1.04
4949 reflections
275 parameters
H atoms treated by a mixture of independent and constrained refinement
Δρ_max_ = 0.71 e Å^−3^
Δρ_min_ = −0.97 e Å^−3^

### 

Data collection: *APEX2* (Bruker, 2009 ▶); cell refinement: *SAINT* (Bruker, 2009 ▶); data reduction: *SAINT*; program(s) used to solve structure: *SHELXTL* (Sheldrick, 2008 ▶); program(s) used to refine structure: *SHELXTL*; molecular graphics: *SHELXTL*; software used to prepare material for publication: *SHELXTL*, *PLATON* (Spek, 2009 ▶) and *publCIF* (Westrip, 2010 ▶).

## Supplementary Material

Crystal structure: contains datablock(s) global, I. DOI: 10.1107/S1600536813021223/sj5348sup1.cif

Structure factors: contains datablock(s) I. DOI: 10.1107/S1600536813021223/sj5348Isup2.hkl

Supplementary material file. DOI: 10.1107/S1600536813021223/sj5348Isup3.cml

Additional supplementary materials:  crystallographic information; 3D view; checkCIF report

## Figures and Tables

**Table 1:** Hydrogen-bond geometry (Å, °)

*D*—H⋯*A*	*D*—H	H⋯*A*	*D*⋯*A*	*D*—H⋯*A*
O4—H1*O*4⋯O3	0.92 (3)	1.67 (3)	2.5337 (14)	156 (3)
O1—H1*O*1⋯O1*W* ^i^	0.86 (2)	1.81 (2)	2.6599 (16)	172.5 (19)
O1*W*—H2*W*1⋯O4^ii^	0.77 (2)	2.13 (2)	2.8756 (16)	166 (2)
O1*W*—H1*W*1⋯O6*A* ^iii^	0.88 (3)	1.93 (3)	2.8078 (17)	175 (3)
O6*A*—H6*A*⋯O1^iii^	0.82 (3)	2.10 (3)	2.8838 (16)	160 (3)
C18*A*—H18*A*⋯O6*A*	0.96	2.44	3.078 (2)	124
C18*A*—H18*C*⋯O4^iv^	0.96	2.60	3.530 (2)	164

Symmetry codes: (i) 
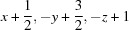
; (ii) 
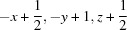
; (iii) 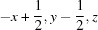; (iv) 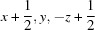.

**Fig. 1.**
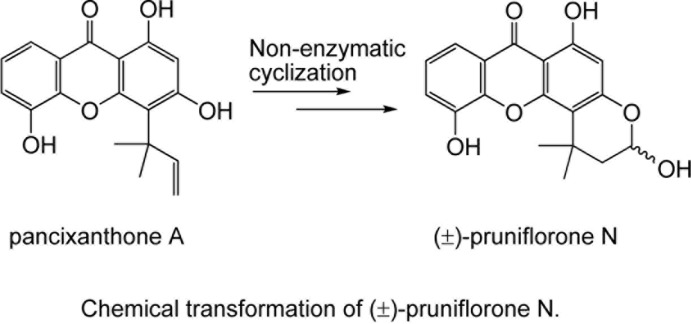
The chemical transformation that yields the title compound.

**Fig. 2.**
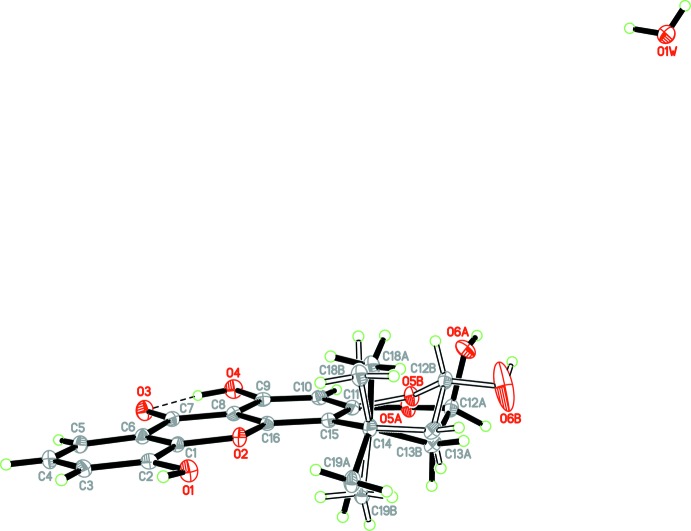
The molecular structure of (I), showing 50% probability displacement ellipsoids and the atom-numbering scheme. O—H···O intramolecular hydrogen bond was drawn as a dashed line. Open bonds show the minor component.

**Fig. 3.**
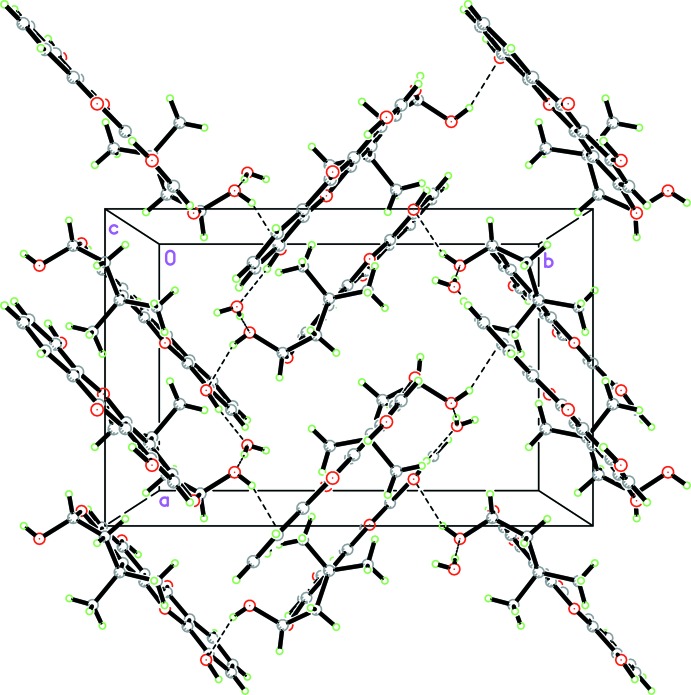
The crystal packing of the major component of (I) viewed along the c axis, showing the three dimensional molecular network. Hydrogen bonds were drawn as dashed lines.

## supplementary crystallographic information

### 1. Comment

Xanthones are reported to exhibit various biological and pharmacological properties (Obolskiy *et al.*, 2009) such as antibacterial (Boonnak, Karalai *et al.*, 2010), antifungal (Gopalakrishnan *et al.*, 1997), anti-inflammatory (Boonnak, Khamthip *et al.*, 2010) and anti-cancer (Ho *et al.*, 2002) activities. We have previously reported several isolated xanthones and their biological activities (Boonnak, Karalai *et al.*, 2010; Boonnak, Khamthip *et al.*, 2010). Among these compounds, the title xanthone (I), which is also known as pruniflorone N, showed antibacterial activity against methicillin-resistant *Staphylococcus aureus* (MRSA) with a MIC value of 9.37 µg mL^-1^. Compound (I) crystallized out in the centrosymmetric *Pbca* space group indicating that the extracted material was a racemate, Figure 1.

Compound (I) has a xanthone nucleus with a pyran ring fused to it in an angular fashion which is rarely found. It crystallized out in a monohydrate form, C_18_H_16_O_6_.H_2_O (Fig. 2). The 2'-hydroxy-4',4'-dimethylpyran ring is disordered over two positions with 0.798 (3):0.202 (3) site occupancies in which the 2'-hydroxy group or the hydroxy groups at atom C12 of the major *A* and minor *B* components were attached in opposite directions. The three ring systems of the xanthone nucleus [C1–C11/C15/C16/O2] are essentially co-planar with an r.m.s. deviation of 0.0270 (1) Å from the plane through all the fourteen non-hydrogen atoms. The O1 and O4 atoms of the two hydroxy substituents also lie close to this plane with deviations of -0.019 (1) and -0.070 (1) Å, respectively. The pyran ring (C11–C15/O5) is in a half-chair conformation with the puckering parameters Q = 0.406 (2) Å, θ = 43.7 (2)° and φ = 250.7 (3)° (Cremer & Pople, 1975) with the puckered C12A and C13A atoms having the deviation of -0.228 (2) and 0.282 (2) Å, respectively for the major component *A* [the corresponding values for the minor component *B* are 0.555 (9) Å, 123.8 (7)° and 33.7 (8)°, and the values for the puckering C12B and C13B atoms are 0.365 (7) and -0.352 (11) Å, respectively]. An intramolecular O4—H1O4···O3 hydrogen bond (Table 1) generates an S(6) ring motif (Bernstein, *et al.*, 1995). The bond distances in (I) are normal (Allen *et al.*, 1987) and comparable to those found in related structures (Boonnak *et al.*, 2006 and Boonnak, Chantrapromma *et al.*, 2010).

The crystal packing of (I) is stabilized by intermolecular O—H···O hydrogen bonds and weak C—H···O interactions (Table 1). The xanthone and water molecules are linked into a three dimensional network by these interactions (Fig. 3). π–π interaction with the distances of Cg_1_···Cg_3_^v^ = 3.6081 (7) Å, Cg_1_···Cg_4_^iv^ = 3.6456 (7) Å and Cg_3_···Cg_4_^iv^ = 3.5982 (7) Å were observed [symmetry code (v) = -1/2+x, y, 1/2-z]; Cg_1_, Cg_3_ and Cg_4_ are the centroids of the C1/C6–C8/C16/O2, C1–C6 and C8–C11/C15/C16 rings, respectively.

### 2. Experimental

The green fruits of *C. formosum* ssp. *pruniflorum* (5.00 kg) were extracted with CH_2_Cl_2_ (2 *x* 20 *L*, for a week) at room temperature and was further evaporated under reduced pressure to afford a crude CH_2_Cl_2_ extract (31.42 g), which was subjected to QCC (Quick Column Chromatography) on silica gel using hexane as a first eluent and then increasing the polarity with acetone to give 14 fractions (F1–F14). Fraction F10 was separated by QCC eluting with a gradient of acetone–hexane to give 17 subfractions (F10A–F10Q). Subfractions F10N was separated by CC and eluted with gradient of EtOAc–hexane to obtain 8 subfractions (F10N1–F10N8). Subfraction F10N6 was separated by CC and eluted with CHCl_3_ to give the title compound as a yellow solid (5.3 mg). Yellow block-shaped single crystals of the title compound suitable for *x*-ray structure determination were recrystallized from acetone–CH_3_OH (9.5:0.5, *v*/*v*) after several days (*M*.p. 523–525 K).

### 3. Refinement

Hydroxy H atoms were located from the difference maps and refined isotropically. The remaining H atoms were placed in calculated positions with d(C—H) = 0.93 Å for aromatic, 0.98 for CH, 0.97 for CH_2_ and 0.96 Å for CH_3_ atoms. The *U*_iso_ values were constrained to be 1.5*U*_eq_ of the carrier atom for methyl H atoms and 1.2*U*_eq_ for the remaining H atoms. A rotating group model was used for the methyl groups. The 2'-hydroxy-4',4'-dimethylpyran is disordered over two sites with refined site occupancies of 0.798 (3) and 0.202 (3). All disordered atoms were subjected to similarity restraints. The same *U_ij_* parameters were used for atom pairs C12A/C12B, C13A/C13B, C18A/C18B, C19A/C19B and O5A/O5B.

### Crystal data

**Table:**

C_18_H_16_O_6_·H_2_O	*D*_x_ = 1.517 Mg m^−^^3^
*M**_r_* = 346.20	Melting point = 523–525 K
Orthorhombic, *P**b**c**a*	Mo *K*α radiation, λ = 0.71073 Å
Hall symbol: -P 2ac 2ab	Cell parameters from 4949 reflections
*a* = 9.8965 (2) Å	θ = 2.0–31.3°
*b* = 15.2329 (3) Å	µ = 0.12 mm^−^^1^
*c* = 20.1122 (4) Å	*T* = 100 K
*V* = 3031.96 (10) Å^3^	Block, yellow
*Z* = 8	0.65 × 0.21 × 0.13 mm
*F*(000) = 1456	

### Data collection

**Table:**

Bruker APEXII CCD area-detector diffractometer	4949 independent reflections
Radiation source: sealed tube	4378 reflections with *I* > 2σ(*I*)
Graphite monochromator	*R*_int_ = 0.030
φ and ω scans	θ_max_ = 31.3°, θ_min_ = 2.0°
Absorption correction: multi-scan (*SADABS*; Bruker, 2009)	*h* = −10→14
*T*_min_ = 0.927, *T*_max_ = 0.985	*k* = −22→22
40070 measured reflections	*l* = −27→29

### Refinement

**Table:**

Refinement on *F*^2^	Primary atom site location: structure-invariant direct methods
Least-squares matrix: full	Secondary atom site location: difference Fourier map
*R*[*F*^2^ > 2σ(*F*^2^)] = 0.052	Hydrogen site location: inferred from neighbouring sites
*wR*(*F*^2^) = 0.145	H atoms treated by a mixture of independent and constrained refinement
*S* = 1.04	*w* = 1/[σ^2^(*F*_o_^2^) + (0.0754*P*)^2^ + 2.1985*P*] where *P* = (*F*_o_^2^ + 2*F*_c_^2^)/3
4949 reflections	(Δ/σ)_max_ = 0.001
275 parameters	Δρ_max_ = 0.71 e Å^−^^3^
0 restraints	Δρ_min_ = −0.97 e Å^−^^3^

### Special details

**Table:**

Experimental. The crystal was placed in the cold stream of an Oxford Cryosystems Cobra open-flow nitrogen cryostat (Cosier & Glazer, 1986) operating at 100.0 (1) K.
Geometry. All e.s.d.'s (except the e.s.d. in the dihedral angle between two l.s. planes) are estimated using the full covariance matrix. The cell e.s.d.'s are taken into account individually in the estimation of e.s.d.'s in distances, angles and torsion angles; correlations between e.s.d.'s in cell parameters are only used when they are defined by crystal symmetry. An approximate (isotropic) treatment of cell e.s.d.'s is used for estimating e.s.d.'s involving l.s. planes.
Refinement. Refinement of *F*^2^ against ALL reflections. The weighted *R*-factor *wR* and goodness of fit *S* are based on *F*^2^, conventional *R*-factors *R* are based on *F*, with *F* set to zero for negative *F*^2^. The threshold expression of *F*^2^ > σ(*F*^2^) is used only for calculating *R*-factors(gt) *etc*. and is not relevant to the choice of reflections for refinement. *R*-factors based on *F*^2^ are statistically about twice as large as those based on *F*, and *R*- factors based on ALL data will be even larger.

### Fractional atomic coordinates and isotropic or equivalent isotropic displacement parameters (Å^2^)

**Table:**

	*x*	*y*	*z*	*U*_iso_*/*U*_eq_	Occ. (<1)
O1	0.58520 (10)	1.15586 (6)	0.38580 (5)	0.01809 (19)	
O2	0.40787 (9)	1.05055 (6)	0.32849 (4)	0.01448 (18)	
O3	0.36164 (10)	1.03467 (6)	0.12623 (5)	0.0205 (2)	
O4	0.17901 (10)	0.92011 (7)	0.14256 (5)	0.0201 (2)	
O5A	0.03637 (14)	0.85070 (10)	0.35563 (7)	0.0161 (3)	0.798 (3)
O6A	0.12745 (13)	0.76392 (8)	0.43976 (6)	0.0202 (3)	0.798 (3)
H6A	0.082 (3)	0.7235 (18)	0.4244 (13)	0.031 (7)*	0.798 (3)
O5B	0.0688 (6)	0.8401 (5)	0.3682 (3)	0.0161 (3)	0.202 (3)
O6B	0.0235 (10)	0.7824 (7)	0.4712 (4)	0.072 (3)	0.202 (3)
H6B	0.0048	0.7334	0.4570	0.108*	0.202 (3)
C1	0.48453 (11)	1.10150 (7)	0.28720 (6)	0.0129 (2)	
C2	0.57959 (12)	1.15648 (8)	0.31807 (6)	0.0144 (2)	
C3	0.66177 (12)	1.20850 (8)	0.27862 (6)	0.0163 (2)	
H3A	0.7261	1.2444	0.2985	0.020*	
C4	0.64937 (13)	1.20774 (8)	0.20928 (6)	0.0179 (2)	
H4A	0.7054	1.2430	0.1835	0.021*	
C5	0.55441 (13)	1.15485 (8)	0.17905 (6)	0.0170 (2)	
H5A	0.5450	1.1553	0.1330	0.020*	
C6	0.47209 (12)	1.10035 (8)	0.21811 (6)	0.0140 (2)	
C7	0.37462 (12)	1.04051 (8)	0.18811 (6)	0.0148 (2)	
C8	0.29411 (12)	0.98915 (7)	0.23357 (6)	0.0135 (2)	
C9	0.19417 (12)	0.93068 (8)	0.20908 (6)	0.0148 (2)	
C10	0.11267 (12)	0.88544 (8)	0.25236 (6)	0.0165 (2)	
H10A	0.0455	0.8483	0.2364	0.020*	
C11	0.13192 (13)	0.89594 (8)	0.32090 (6)	0.0164 (2)	
C14	0.25267 (13)	0.95266 (8)	0.42430 (6)	0.0174 (2)	
C15	0.23195 (12)	0.94999 (8)	0.34905 (6)	0.0142 (2)	
C16	0.31063 (11)	0.99645 (7)	0.30282 (6)	0.0125 (2)	
C12A	0.05298 (16)	0.84187 (10)	0.42636 (8)	0.0164 (3)	0.798 (3)
H12A	−0.0370	0.8351	0.4461	0.020*	0.798 (3)
C13A	0.11699 (19)	0.92194 (15)	0.45651 (13)	0.0174 (4)	0.798 (3)
H13A	0.1332	0.9104	0.5033	0.021*	0.798 (3)
H13B	0.0528	0.9700	0.4538	0.021*	0.798 (3)
C18A	0.36942 (19)	0.89084 (13)	0.44175 (10)	0.0194 (4)	0.798 (3)
H18A	0.3486	0.8326	0.4266	0.029*	0.798 (3)
H18B	0.3823	0.8902	0.4891	0.029*	0.798 (3)
H18C	0.4506	0.9109	0.4205	0.029*	0.798 (3)
C19A	0.28024 (18)	1.04439 (18)	0.45227 (13)	0.0197 (4)	0.798 (3)
H19A	0.2176	1.0855	0.4333	0.030*	0.798 (3)
H19B	0.3709	1.0617	0.4414	0.030*	0.798 (3)
H19C	0.2695	1.0436	0.4997	0.030*	0.798 (3)
C12B	0.1244 (7)	0.8194 (4)	0.4320 (3)	0.0164 (3)	0.202 (3)
H12B	0.2063	0.7834	0.4295	0.020*	0.202 (3)
C13B	0.1485 (10)	0.9088 (8)	0.4616 (6)	0.0174 (4)	0.202 (3)
H13C	0.0658	0.9430	0.4602	0.021*	0.202 (3)
H13D	0.1759	0.9031	0.5077	0.021*	0.202 (3)
C18B	0.3963 (9)	0.9160 (6)	0.4462 (5)	0.0194 (4)	0.202 (3)
H18D	0.4651	0.9575	0.4342	0.029*	0.202 (3)
H18E	0.4132	0.8612	0.4241	0.029*	0.202 (3)
H18F	0.3975	0.9072	0.4934	0.029*	0.202 (3)
C19B	0.2419 (10)	1.0488 (9)	0.4550 (7)	0.0197 (4)	0.202 (3)
H19D	0.1551	1.0733	0.4446	0.030*	0.202 (3)
H19E	0.3115	1.0852	0.4365	0.030*	0.202 (3)
H19F	0.2526	1.0458	0.5024	0.030*	0.202 (3)
O1W	0.28541 (14)	0.24213 (8)	0.57159 (6)	0.0341 (3)	
H1O4	0.239 (3)	0.9598 (17)	0.1248 (14)	0.057 (8)*	
H1O1	0.654 (2)	1.1869 (15)	0.3967 (11)	0.037 (6)*	
H2W1	0.307 (2)	0.1986 (15)	0.5874 (11)	0.032 (5)*	
H1W1	0.316 (3)	0.2464 (17)	0.5304 (14)	0.053 (7)*	

### Atomic displacement parameters (Å^2^)

**Table:**

	*U*^11^	*U*^22^	*U*^33^	*U*^12^	*U*^13^	*U*^23^
O1	0.0202 (4)	0.0208 (4)	0.0132 (4)	−0.0044 (3)	−0.0025 (3)	−0.0005 (3)
O2	0.0139 (4)	0.0180 (4)	0.0115 (4)	−0.0039 (3)	−0.0002 (3)	0.0012 (3)
O3	0.0252 (5)	0.0249 (5)	0.0114 (4)	−0.0025 (4)	−0.0014 (3)	−0.0001 (3)
O4	0.0225 (5)	0.0230 (4)	0.0148 (4)	−0.0026 (4)	−0.0040 (3)	−0.0032 (3)
O5A	0.0150 (7)	0.0206 (6)	0.0127 (7)	−0.0041 (5)	−0.0042 (4)	0.0038 (4)
O6A	0.0239 (6)	0.0134 (5)	0.0232 (6)	−0.0028 (4)	−0.0061 (5)	0.0020 (4)
O5B	0.0150 (7)	0.0206 (6)	0.0127 (7)	−0.0041 (5)	−0.0042 (4)	0.0038 (4)
O6B	0.067 (6)	0.103 (7)	0.046 (4)	−0.054 (5)	−0.018 (4)	0.041 (5)
C1	0.0124 (5)	0.0137 (5)	0.0128 (5)	0.0005 (4)	0.0012 (4)	0.0012 (4)
C2	0.0142 (5)	0.0139 (5)	0.0151 (5)	0.0014 (4)	−0.0001 (4)	−0.0005 (4)
C3	0.0158 (5)	0.0139 (5)	0.0191 (5)	−0.0016 (4)	0.0007 (4)	0.0000 (4)
C4	0.0189 (5)	0.0166 (5)	0.0181 (5)	−0.0014 (4)	0.0043 (4)	0.0026 (4)
C5	0.0187 (5)	0.0175 (5)	0.0147 (5)	0.0005 (4)	0.0026 (4)	0.0017 (4)
C6	0.0143 (5)	0.0150 (5)	0.0126 (5)	0.0009 (4)	0.0007 (4)	0.0005 (4)
C7	0.0155 (5)	0.0157 (5)	0.0132 (5)	0.0020 (4)	−0.0004 (4)	0.0001 (4)
C8	0.0131 (5)	0.0145 (5)	0.0127 (5)	0.0010 (4)	−0.0008 (4)	−0.0004 (4)
C9	0.0148 (5)	0.0148 (5)	0.0149 (5)	0.0024 (4)	−0.0026 (4)	−0.0022 (4)
C10	0.0147 (5)	0.0148 (5)	0.0201 (6)	−0.0007 (4)	−0.0005 (4)	−0.0033 (4)
C11	0.0167 (5)	0.0134 (5)	0.0191 (6)	−0.0004 (4)	0.0034 (4)	−0.0008 (4)
C14	0.0193 (5)	0.0190 (5)	0.0137 (5)	0.0008 (4)	0.0034 (4)	0.0033 (4)
C15	0.0151 (5)	0.0130 (5)	0.0145 (5)	0.0009 (4)	0.0017 (4)	0.0002 (4)
C16	0.0116 (5)	0.0125 (4)	0.0133 (5)	0.0005 (4)	−0.0007 (4)	0.0001 (4)
C12A	0.0149 (6)	0.0202 (7)	0.0142 (6)	−0.0004 (5)	0.0013 (5)	0.0024 (5)
C13A	0.0145 (11)	0.0199 (9)	0.0178 (7)	0.0005 (7)	0.0024 (8)	−0.0019 (6)
C18A	0.0179 (9)	0.0230 (10)	0.0172 (7)	−0.0029 (6)	−0.0011 (6)	0.0038 (7)
C19A	0.0226 (11)	0.0229 (7)	0.0137 (6)	−0.0067 (11)	0.0018 (10)	−0.0021 (5)
C12B	0.0149 (6)	0.0202 (7)	0.0142 (6)	−0.0004 (5)	0.0013 (5)	0.0024 (5)
C13B	0.0145 (11)	0.0199 (9)	0.0178 (7)	0.0005 (7)	0.0024 (8)	−0.0019 (6)
C18B	0.0179 (9)	0.0230 (10)	0.0172 (7)	−0.0029 (6)	−0.0011 (6)	0.0038 (7)
C19B	0.0226 (11)	0.0229 (7)	0.0137 (6)	−0.0067 (11)	0.0018 (10)	−0.0021 (5)
O1W	0.0452 (7)	0.0290 (6)	0.0280 (6)	0.0157 (5)	0.0178 (5)	0.0078 (5)

### Geometric parameters (Å, º)

**Table:**

O1—C2	1.3635 (15)	C11—C15	1.4065 (17)
O1—H1O1	0.86 (2)	C14—C13B	1.439 (12)
O2—C1	1.3665 (14)	C14—C15	1.5279 (17)
O2—C16	1.3681 (14)	C14—C19A	1.531 (3)
O3—C7	1.2543 (15)	C14—C18A	1.531 (2)
O4—C9	1.3557 (15)	C14—C13A	1.563 (3)
O4—H1O4	0.92 (3)	C14—C18B	1.589 (10)
O5A—C11	1.3627 (18)	C14—C19B	1.593 (14)
O5A—C12A	1.4383 (19)	C15—C16	1.4041 (16)
O6A—C12A	1.4233 (19)	C12A—C13A	1.502 (3)
O6A—H6A	0.82 (3)	C12A—H12A	0.9800
O5B—C11	1.421 (7)	C13A—H13A	0.9700
O5B—C12B	1.432 (8)	C13A—H13B	0.9700
O6B—C12B	1.391 (10)	C18A—H18A	0.9600
O6B—H6B	0.8200	C18A—H18B	0.9600
C1—C6	1.3951 (15)	C18A—H18C	0.9600
C1—C2	1.4043 (16)	C19A—H19A	0.9600
C2—C3	1.3852 (16)	C19A—H19B	0.9600
C3—C4	1.4001 (17)	C19A—H19C	0.9600
C3—H3A	0.9300	C12B—C13B	1.506 (14)
C4—C5	1.3791 (18)	C12B—H12B	0.9800
C4—H4A	0.9300	C13B—H13C	0.9700
C5—C6	1.4037 (16)	C13B—H13D	0.9700
C5—H5A	0.9300	C18B—H18D	0.9600
C6—C7	1.4579 (17)	C18B—H18E	0.9600
C7—C8	1.4432 (16)	C18B—H18F	0.9600
C8—C16	1.4068 (16)	C19B—H19D	0.9600
C8—C9	1.4192 (16)	C19B—H19E	0.9600
C9—C10	1.3723 (17)	C19B—H19F	0.9600
C10—C11	1.4007 (17)	O1W—H2W1	0.76 (2)
C10—H10A	0.9300	O1W—H1W1	0.88 (3)
			
C2—O1—H1O1	106.5 (15)	C15—C14—C19B	113.5 (5)
C1—O2—C16	120.22 (9)	C18A—C14—C19B	121.8 (4)
C9—O4—H1O4	103.5 (17)	C13A—C14—C19B	93.3 (4)
C11—O5A—C12A	118.35 (12)	C18B—C14—C19B	106.0 (5)
C12A—O6A—H6A	105.8 (19)	C16—C15—C11	114.76 (11)
C11—O5B—C12B	124.3 (5)	C16—C15—C14	124.61 (11)
C12B—O6B—H6A	65.6 (13)	C11—C15—C14	120.58 (11)
C12B—O6B—H6B	109.5	O2—C16—C15	116.35 (10)
O2—C1—C6	123.31 (10)	O2—C16—C8	120.19 (10)
O2—C1—C2	116.24 (10)	C15—C16—C8	123.45 (11)
C6—C1—C2	120.46 (11)	O6A—C12A—O5A	108.92 (13)
O1—C2—C3	123.52 (11)	O6A—C12A—C13A	112.50 (13)
O1—C2—C1	117.70 (10)	O5A—C12A—C13A	111.81 (15)
C3—C2—C1	118.78 (11)	O6A—C12A—H12A	107.8
C2—C3—C4	120.95 (11)	O5A—C12A—H12A	107.8
C2—C3—H3A	119.5	C13A—C12A—H12A	107.8
C4—C3—H3A	119.5	C12A—C13A—C14	115.99 (17)
C5—C4—C3	120.25 (11)	C12A—C13A—H13A	108.3
C5—C4—H4A	119.9	C14—C13A—H13A	108.3
C3—C4—H4A	119.9	C12A—C13A—H13B	108.3
C4—C5—C6	119.62 (11)	C14—C13A—H13B	108.3
C4—C5—H5A	120.2	H13A—C13A—H13B	107.4
C6—C5—H5A	120.2	C14—C18A—H18A	109.5
C1—C6—C5	119.93 (11)	C14—C18A—H18B	109.5
C1—C6—C7	118.58 (11)	C14—C18A—H18C	109.5
C5—C6—C7	121.48 (11)	C14—C19A—H19A	109.5
O3—C7—C8	122.25 (11)	C14—C19A—H19B	109.5
O3—C7—C6	121.51 (11)	C14—C19A—H19C	109.5
C8—C7—C6	116.23 (10)	O6B—C12B—O5B	108.8 (6)
C16—C8—C9	118.31 (11)	O6B—C12B—C13B	104.9 (8)
C16—C8—C7	121.35 (10)	O5B—C12B—C13B	102.5 (7)
C9—C8—C7	120.33 (10)	O6B—C12B—H6A	58.2 (11)
O4—C9—C10	120.09 (11)	O5B—C12B—H6A	90.9 (11)
O4—C9—C8	119.61 (11)	C13B—C12B—H6A	161.6 (12)
C10—C9—C8	120.30 (11)	O6B—C12B—H12B	113.3
C9—C10—C11	119.15 (11)	O5B—C12B—H12B	113.3
C9—C10—H10A	120.4	C13B—C12B—H12B	113.3
C11—C10—H10A	120.4	H6A—C12B—H12B	71.7
O5A—C11—C10	110.63 (11)	C14—C13B—C12B	109.1 (8)
O5A—C11—C15	125.32 (12)	C14—C13B—H13C	109.9
C10—C11—C15	123.96 (11)	C12B—C13B—H13C	109.9
C10—C11—O5B	122.0 (3)	C14—C13B—H13D	109.9
C15—C11—O5B	113.0 (3)	C12B—C13B—H13D	109.9
C13B—C14—C15	114.1 (5)	H13C—C13B—H13D	108.3
C13B—C14—C19A	111.1 (5)	C14—C18B—H18D	109.5
C15—C14—C19A	114.34 (14)	C14—C18B—H18E	109.5
C13B—C14—C18A	97.8 (4)	H18D—C18B—H18E	109.5
C15—C14—C18A	108.17 (12)	C14—C18B—H18F	109.5
C19A—C14—C18A	110.04 (14)	H18D—C18B—H18F	109.5
C15—C14—C13A	106.70 (13)	H18E—C18B—H18F	109.5
C19A—C14—C13A	105.91 (13)	C14—C19B—H19D	109.5
C18A—C14—C13A	111.67 (12)	C14—C19B—H19E	109.5
C13B—C14—C18B	109.5 (5)	H19D—C19B—H19E	109.5
C15—C14—C18B	112.6 (4)	C14—C19B—H19F	109.5
C19A—C14—C18B	93.4 (3)	H19D—C19B—H19F	109.5
C13A—C14—C18B	123.3 (3)	H19E—C19B—H19F	109.5
C13B—C14—C19B	100.2 (6)	H2W1—O1W—H1W1	111 (2)
			
C16—O2—C1—C6	−1.71 (17)	O5B—C11—C15—C14	−6.8 (3)
C16—O2—C1—C2	178.53 (10)	C13B—C14—C15—C16	172.2 (5)
O2—C1—C2—O1	−1.42 (16)	C19A—C14—C15—C16	42.85 (17)
C6—C1—C2—O1	178.81 (10)	C18A—C14—C15—C16	−80.14 (15)
O2—C1—C2—C3	178.89 (10)	C13A—C14—C15—C16	159.59 (13)
C6—C1—C2—C3	−0.87 (17)	C18B—C14—C15—C16	−62.2 (4)
O1—C2—C3—C4	−178.60 (11)	C19B—C14—C15—C16	58.3 (4)
C1—C2—C3—C4	1.06 (18)	C13B—C14—C15—C11	−10.3 (5)
C2—C3—C4—C5	0.05 (19)	C19A—C14—C15—C11	−139.67 (13)
C3—C4—C5—C6	−1.34 (19)	C18A—C14—C15—C11	97.34 (14)
O2—C1—C6—C5	179.85 (11)	C13A—C14—C15—C11	−22.94 (16)
C2—C1—C6—C5	−0.40 (18)	C18B—C14—C15—C11	115.3 (3)
O2—C1—C6—C7	−1.39 (17)	C19B—C14—C15—C11	−124.2 (4)
C2—C1—C6—C7	178.36 (10)	C1—O2—C16—C15	−176.75 (10)
C4—C5—C6—C1	1.51 (18)	C1—O2—C16—C8	3.63 (16)
C4—C5—C6—C7	−177.21 (11)	C11—C15—C16—O2	179.21 (10)
C1—C6—C7—O3	−177.90 (11)	C14—C15—C16—O2	−3.18 (17)
C5—C6—C7—O3	0.84 (18)	C11—C15—C16—C8	−1.18 (17)
C1—C6—C7—C8	2.41 (16)	C14—C15—C16—C8	176.43 (11)
C5—C6—C7—C8	−178.85 (11)	C9—C8—C16—O2	178.52 (10)
O3—C7—C8—C16	179.76 (11)	C7—C8—C16—O2	−2.47 (17)
C6—C7—C8—C16	−0.56 (16)	C9—C8—C16—C15	−1.07 (17)
O3—C7—C8—C9	−1.26 (18)	C7—C8—C16—C15	177.93 (11)
C6—C7—C8—C9	178.42 (10)	C11—O5A—C12A—O6A	−90.38 (16)
C16—C8—C9—O4	−177.83 (10)	C11—O5A—C12A—C13A	34.57 (19)
C7—C8—C9—O4	3.16 (17)	O6A—C12A—C13A—C14	70.0 (2)
C16—C8—C9—C10	2.57 (17)	O5A—C12A—C13A—C14	−52.97 (19)
C7—C8—C9—C10	−176.45 (11)	C13B—C14—C13A—C12A	−79 (2)
O4—C9—C10—C11	178.68 (11)	C15—C14—C13A—C12A	45.45 (18)
C8—C9—C10—C11	−1.72 (18)	C19A—C14—C13A—C12A	167.66 (16)
C12A—O5A—C11—C10	169.91 (13)	C18A—C14—C13A—C12A	−72.56 (19)
C12A—O5A—C11—C15	−13.5 (2)	C18B—C14—C13A—C12A	−87.2 (4)
C12A—O5A—C11—O5B	37.4 (11)	C19B—C14—C13A—C12A	161.2 (5)
C9—C10—C11—O5A	175.95 (12)	C11—O5B—C12B—O6B	165.2 (8)
C9—C10—C11—C15	−0.73 (19)	C11—O5B—C12B—C13B	54.5 (9)
C9—C10—C11—O5B	−168.5 (3)	C15—C14—C13B—C12B	48.6 (7)
C12B—O5B—C11—O5A	−154.9 (17)	C19A—C14—C13B—C12B	179.6 (5)
C12B—O5B—C11—C10	150.7 (5)	C18A—C14—C13B—C12B	−65.3 (6)
C12B—O5B—C11—C15	−18.3 (8)	C13A—C14—C13B—C12B	109 (3)
O5A—C11—C15—C16	−174.07 (12)	C18B—C14—C13B—C12B	−78.6 (8)
C10—C11—C15—C16	2.13 (17)	C19B—C14—C13B—C12B	170.3 (7)
O5B—C11—C15—C16	170.9 (3)	O6B—C12B—C13B—C14	179.8 (7)
O5A—C11—C15—C14	8.22 (19)	O5B—C12B—C13B—C14	−66.7 (7)
C10—C11—C15—C14	−175.59 (11)		

### Hydrogen-bond geometry (Å, º)

**Table:**

*D*—H···*A*	*D*—H	H···*A*	*D*···*A*	*D*—H···*A*
O4—H1*O*4···O3	0.92 (3)	1.67 (3)	2.5337 (14)	156 (3)
O1—H1*O*1···O1*W*^i^	0.86 (2)	1.81 (2)	2.6599 (16)	172.5 (19)
O1*W*—H2*W*1···O4^ii^	0.77 (2)	2.13 (2)	2.8756 (16)	166 (2)
O1*W*—H1*W*1···O6*A*^iii^	0.88 (3)	1.93 (3)	2.8078 (17)	175 (3)
O6*A*—H6*A*···O1^iii^	0.82 (3)	2.10 (3)	2.8838 (16)	160 (3)
C18*A*—H18*A*···O6*A*	0.96	2.44	3.078 (2)	124
C18*A*—H18*C*···O4^iv^	0.96	2.60	3.530 (2)	164

Symmetry codes: (i) *x*+1/2, −*y*+3/2, −*z*+1; (ii) −*x*+1/2, −*y*+1, *z*+1/2; (iii) −*x*+1/2, *y*−1/2, *z*; (iv) *x*+1/2, *y*, −*z*+1/2.
